# Dr. Mohr’s Case of Cancer

**Published:** 1889-02

**Authors:** C. Mohr


### DR. MOHR'S CASE OF CANCER.

Messrs. Editors:—You will do me the favor to set me right before your readers. In the December number, your report concerning the case of carcinoma I related to the Homœpathic Medical Society of Pennsylvania, is far from correct. You say: "The patient, a woman, was being treated with Arsenic. Erysipelas broke out and cured the cancer. This accidental cure, Dr. Mohr declared has possibly opened up a way by which cancer can be cured by the virus of erysipelas. * * * Dr. Mohr's report illustrates the haste physicians make to form *theories*; presuming upon this case, Dr. Mohr thinks the virus of erysipelas may prove a specific for cancer."

I made no declarations, formed no theories, but related simple clinical facts, and propounded a question to elicit discussion. My paper was published in full in the *N. A. Journal of Homœopathy* for November. If you will read it, you will find that the conduct of the case shows good homœopathic treatment, and that *Arsenicum* was prescribed *after* the supervention of erysipelas on well-known indications.

Yours respectfully,

C. Mohr.

---

[We have no desire to misrepresent Dr. Mohr, and are very sorry if we have inadvertently done so.

But, to show that we really did *not* misrepresent him, we quote from the closing paragraph of his paper. The Doctor writes: "I am not quite satisfied that the erysipelas alone cured the cancer; the Arsenicum may have had something to do with the result. * * * And now for an important question: In the light of this case, would one be justified in inoculating a patient suffering from cancer, where operative measures were contra-indicated, with the virus of erysipelas, to bring about a possible obliteration of the neoplasm?"

Dr. Mohr does not consider the asking of this "important question," a method of suggesting a theory! And a very hastily formed theory at that!—EDITORS.]

